# Environmental Cadmium Exposure Promotes the Development, Progression and Chemoradioresistance of Esophageal Squamous Cell Carcinoma

**DOI:** 10.3389/fcell.2022.792933

**Published:** 2022-02-18

**Authors:** Jiongyu Chen, Zhangzhu Zhou, Xueqiong Lin, Jiahui Liao, Yujie Zhang, Bingmeng Xie, Yiteng Huang, Lin Peng

**Affiliations:** ^1^ Central Laboratory, Cancer Hospital of Shantou University Medical College, Shantou, China; ^2^ Department of Laboratory, Jiangmen Central Hospital, Jiangmen, China; ^3^ Department of Laboratory Medicine, Cancer Hospital of Shantou University Medical College, Shantou, China; ^4^ Department of Chemical Engineering, Guangdong Technion-Israel Institute of Technology, Shantou, China; ^5^ Health Care Center, The First Affiliated Hospital of Shantou University Medical College, Shantou, China

**Keywords:** cadmium, esophageal squamous cell carcinoma, chemoradioresistance, cancer stem cell, Wnt/β-catenin

## Abstract

Cadmium (Cd) exposure has been implicated in the etiology of esophageal squamous cell carcinoma (ESCC), albeit with inconsistent results from epidemiologic studies and without causal evidence. In this study, we explore the relationship of Cd exposure and the development, progression and therapeutic resistance of ESCC. A total of 150 ESCC patients and 177 matched controls from a coastal region with a high incidence of ESCC in China were included in the study. It was found that the median blood Cd level (BCL) was significantly higher in ESCC patients than that in the controls. Odds ratios for ESCC risk were 3.12 (95% CI 1.54-6.30) and 3.71 (95% CI 1.84-7.48) in the third and fourth quartiles of Cd distribution, respectively. Notably, BCL above 4.71 μg/L was strongly associated with shorter progression-free survival time compared to that below 1.60 μg/L (*p* < 0.001). The chronic Cd-treated ESCC cells (CCT-ESCC) CCT-EC109 and CCT-EC9706 exhibited increased cell proliferation and tumorigenesis, enhanced migration and invasion, and upregulated EMT biomarkers following 12 weeks of exposure to 5 μM cadmium chloride. Furthermore, Cd treatment attenuated the efficacy of 5-fluorouracil, cisplatin and irradiation treatment in CCT-ESCC cells both *in vitro* and *in vivo*. Moreover, we revealed that Cd stimulated the cancer cell stemness and Wnt/β-catenin signaling pathway in the CCT-ESCC cells. Additionally, 5-aza-2-deoxy-cytidine treatment resulted in suppression of the Wnt/β-catenin signaling pathway and rescue of the Cd-induced cell radioresistance. These results offer new insights into the role of environmental Cd exposure in the development, progression and chemoradioresistance of ESCC.

## Introduction

Esophageal cancer is a malignant digestive tract cancer which ranks the seventh in incidence and is the sixth leading cause of cancer-related mortality (Sung et al., 2021). Esophageal squamous cell carcinoma (ESCC) accounts for 90% of all cases of esophageal cancer globally (Abnet et al., 2018). Therefore, understanding the etiology and tumor biology, as well as identifying risk factors for ESCC progression and therapeutic resistance, are of great importance.

Some epidemiological features, such as the varied ESCC incidence in gender and geographical regions, point to the possible role of environmental factors in the development of ESCC. Heavy drinking and smoking and their synergistic effects are documented to be the major risk factors of ESCC in western countries. However, in lower income countries with a high-incidence of ESCC, the major risk factors for ESCC have yet to be elucidated, although dietary components (e.g., nutritional deficiencies or nitrosamines consumption) and environmental carcinogen exposure have been suspected (Ke et al., 2002; Li and Yu, 2003; Lin et al., 2017; McCormack et al., 2017; Smyth et al., 2017; Znaor et al., 2003). The definitive environmental carcinogen with strong evidence for a causative role in ESCC has not been established. The individual variability, which includes genes and lifestyle of each person, has to be taken into account in precision cancer medicine (Collins and Varmus, 2015). Nevertheless, little attention has been paid to the potential antagonistic effect of environmental factors in the therapeutic efficacy of cancer treatment.

Cadmium (Cd) is a ubiquitous environmental pollutant, whose biological half-life in humans is 10–35 years, thereby posing long-term health risks. Cd and its compounds have been designated as Group Ⅰcarcinogens since 1993, by the International Agency for Research on Cancer (IARC), based on the strong epidemiological evidence that showed a causal connection with the development of lung cancer (IARC, 1993). Later, a positive association between Cd exposure and cancer of the kidney and prostate in human was documented (Humans, 2012). However, the association between Cd exposure and ESCC remains equivocal, which is subjected to further verification in clinical and experimental studies. Interestingly, an earlier preliminary study demonstrated that Cd prevented the cytotoxic effect of 5-fluorouracil (5-FU) on breast cancer cells by modifying the cell cycle and apoptotic profiles (Asara et al., 2012). Whether Cd could also cause an adverse impact on the sensitivity of ESCC cells to chemotherapeutic agents, and irradiation treatment, remains unknown.

In this study, we conducted a case-control study to explore the association between the risk and prognosis of ESCC and environmental Cd exposure in a general population from a region with a high incidence of ESCC. This area displayed 11.43 ESCC patients per 100,000 compared to the worldwide incidence of 5.2 per 100,000 (Tian et al., 2020). Furthermore, we investigated the cause-and-effect relationship between chronic Cd exposure and malignant progression and chemoradiosensitivity of ESCC cells both *in vitro* and *in vivo*. Lastly, we investigated the possible molecular mechanisms behind Cd carcinogenesis and chemoradioresistance in ESCC.

## Materials and Methods

### Case-Control Study

#### Study Population and Sample Collection

The case-control study was carried out in the Cancer Hospital and the First Affiliated Hospital of Shantou University Medical College. One hundred and fifty ESCC patients with an average age of 59 ± 10 years and 177 healthy controls with 57.47 ± 14.12 years old were included in the study. The cases were confirmed histopathologically and followed chemoradiotherapy. The inclusion criteria for the patients and controls were as follows: over 18 years old, of Teochew origin and living in the Chaoshan region for at least 10 years. Exclusion criteria were pregnancy, occupational Cd exposure history and a recent history of blood transfusion. Since the patients and controls have similar dietary style and lifestyle, they were matched primarily by frequency of geographic and social class status. A sample of 2 ml venous whole blood was collected from every participant and stored in K_2_-EDTA metal-free vials at a −70°C refrigerator until analysis. All participants gave their written informed consents after receiving detailed explanations of the study and potential consequences prior to enrollment. This study was approved by the Ethical Committee of the Cancer Hospital of Shantou University Medical College.

#### Blood Sample Processing and Cd Level Analysis

Before analysis, blood samples were digested as previously described (Peng et al., 2015). A multi-element calibration standard solution (10 μg/ml, Agilent Technologies) was used to establish the standard curve, then the Cd content was determined by an Agilent 7900 ICP-MS (Agilent Technologies, Santa Monica, CA, United States) with 99.999% Argon. The linear correlation coefficient of the standard calibration curve was 0.9997. Multiple-element standard stock solutions (100 μg/ml, Agilent Technologies) were used as internal standards and the trace elements of the whole blood (Seronorm, Billingstad, Norway) was determined for daily internal quality control. The limit of detection was 0.001 μg Cd/L.

### *In Vitro* Study

#### Cell Lines and Culture

The human ESCC cell lines EC109 and EC9706 were gifts from Dr. Jing Yu and Dr. Xianbin (Department of Gastroenterology, The First Affiliated Hospital of Shantou University Medical College, China) (Peng et al., 2020; Yu et al., 2019). Cells were maintained in RPMI-1640 medium (HIMEDIA, Mumbai, India) with 10% fetal bovine serum (FBS; HyClone, Logan, UT, United States) in an atmosphere of 5% CO_2_ at 37°C. To simulate environmental Cd exposure, 5 μM cadmium chloride (purity 99%; Sigma, St. Louis, MO, United States) was selected for continuous exposure based on previous studies (Fujiki et al., 2017; Peng et al., 2019; Son et al., 2012; Wei and Shaikh, 2017) and the LC_50_ values. ESCC cells that were chronically treated (over 12 weeks) with Cd were named CCT-ESCC, specifically CCT-EC109 and CCT-EC9706 cells.

#### Cell Proliferation and Cytotoxicity Evaluation

Cell viability was quantitatively analyzed by 3-(4,5-dimethylthiazol-2yl)-2,5-diphenyltetrazolium bromide (MTT) assay (Sigma–Aldrich, St. Louis, MO, United States) using a Multiskan MK3 reader (Thermo Fisher Scientific, Inc.) as described previously (Peng et al., 2019). To determine cell survival, the value of relative cell viability for chemosensitivity analysis was normalized to cells without drug treatment, which was equivalent to 100% cell viability. Experiments were performed in triplicate.

#### Cell Migration and Invasion Assay

Transwell assays were used to analyze the influence of Cd on cell migration and invasion ability. The procedures have previously been described in detail (Peng et al., 2019). Experiments were independently performed in triplicate.

#### Drug and Irradiation Treatment

To investigate the response of CCT-ESCC cells to anti-cancer drugs, both CCT-ESCC cells and their parental cells were treated with 0, 0.5, 1, 5, 10, 20 μg/ml of 5-fluorouracil (5-FU) (Hainan Choitec Pharmaceuticals Co., Ltd., Hainan, China) and 0, 0.1, 0.5, 1, 2.5, 5, 10 μg/ml of cisplatin (DDP, Hansoh Pharma Co. LTD., Jiangsu, China) for 48 h. To explore the role of DNA methylation in Wnt/β-catenin signaling and in Cd-induced radio-resistance, cells were treated with 50 mM 5-aza-2-deoxy-cytidine (5-aza-CdR, Santa Cruz, Dallas, Texas, United States), a demethylation agent for 48 h. For irradiation experiment, the cells were subjected to 6 MV X-ray irradiation using a linear accelerator (23EX; Varian, United States) at single doses of 0, 2, 4, 6 and 8 Gy with the following radiation characteristics: size of the radiation field, 30 cm × 30 cm; the source skin distance, 100 cm; and dose-rate, 285 cGy/min.

#### Clonogenic Cell Survival Assay

After X-ray radiation, the cells were cultured for 14 days, and the colonies were stained with Giemsa for 10 min following paraformaldehyde fixation. The number of colonies (≥50 cells per colony) was counted under a light microscope. The radiosensitivity was estimated in the form of percentage of survival fraction (SF) and the sensitization enhancement ratio (SER) was calculated by cell survival curve using the multi-target/single-hit model as previously described (Cai et al., 2019). All groups were assessed in triplicate.

#### Tumor Sphere Formation Assay

The tumor sphere formation assay was used to estimate the percentage of cancer stem/progenitor cells present in EC109, EC9706, CCT-EC109 and CCT-EC9706 cells according to a method previously described (Johnson et al., 2013). The sphere number (>50 cells) in each well was quantified from each replicate well under a microscope (Olympus, Japan).

#### Flow Cytometry Assay

The CCT-EC109 and CCT-EC9706 cells and their parental cells were measured for cells expressing high levels of aldehyde dehydrogenase (ALDH), using an ALDEFLUOR Kit (STEMCELL Technologies, Canada) according to the manufacturer’s protocol. Furthermore, cells were treated with CD44 monoclonal antibody-PE conjugates and CD24 monoclonal antibody labeled with FITC (Becton Dickinson and Company, United States) for 2 h at room temperature. The flow cytometric analysis for CSC identification was performed using the C6 Flow Cytometer (Becton, Dickinson and Company, United States).

#### Western Blotting

Western blot was carried out according to standard procedures, as described previously (Peng et al., 2019). The following antibodies were used for EMT analysis: antibody against β-catenin, N-cadherin, vimentin and E-cadherin (1:1,000; Cell Signaling Technology, Beverly, MA, United States). The E/N cadherin switch was therefore evaluated with the E/N cadherin expression ratio.

#### Immunofluorescence Analysis

Immunofluorescence detection was performed for β-catenin and pGSK3β using primary anti-β-catenin (1:100, Cell Signaling Technology, United States), pGSK3β (1:100, Cell signaling Technology, United States) and secondary antibodies (1:2000, ZSGB-BIO, China) according to previously described methods (Peng et al., 2019). Nuclei were stained with DAPI. Fluorescence images were taken with a fluorescence microscope (Olympus BX51, Japan).

#### Quantitative Real-Time PCR

Total RNA was extracted from the cells using TRIzol (Invitrogen, Grand Island, NY, United States) and reversely transcribed using a reverse transcriptase PCR kit (Takara, Shiga, Japan). qPCR was performed using SYBR Green qPCR SuperMix (Bio-Rad, California, United States) and an ABI 7500 Fast Sequence Detection system (ABI, Foster, United States). The following primers were used for qPCR analysis for β-catenin signaling: *cyclin D1* forward 5-TGT​CCT​ACT​ACC​GCC​TCA​CA-3 and reverse 5-CAG​GGC​TTC​GAT​CTG​CTC-3; *cyclin E* forward 5-AAA​AGG​TTT​CAG​GGT​ATC​AG-3 and reverse 5- TGT​GGG​TCT​GTA​TGT​TGT​G-3; *c-myc* forward 5-GCC​CCT​CAA​CGT​TAG​CTT​CA -3 and reverse 5- TTC​CAG​ATA​TCC​TCG​CTG​GG-3; *c-jun* forward 5-AAG​AAC​TCG​GAC​CTC​CTC​AC-3 and reverse 5- CTC​CTG​CTC​ATC​TGT​CAC​G-3; *β-actin* forward 5- AGC​GAG​CAT​CCC​CCA​AAG​TT -3 and reverse 5-GGG​CAC​GAA​GGC​TCA​TCA​TT-3. Gene expression relative to β-actin was determined by the comparative CT method (2^−ΔΔCT^). All experiments were performed in triplicate.

### *In Vivo* Analysis

#### Xenograft Tumor Model

The study was conducted on 4-week-old BALB/c nude mice under pathogen-free conditions. The mice were obtained from Vital River (Beijing, People’s Republic of China) and acclimatized for a week prior to use in the study. Xenografts were established by subcutaneous injection with 100 µl of EC109 or CCT-EC109 cells (2×10^6^ cells/ml) into the left axilla according to our previous methods (Peng et al., 2019). The growth curve of the xenograft tumor was generated as a function of the volume of the tumor (Volume = width^2^ × length × 0.5). On day 47 after tumor cell injection, the mice were euthanized and the tumors were excised and weighed. All experimental procedures were approved by the Animal Ethics Committee of Shantou University Medical College and followed the guidelines of the Animal Laboratory Center.

#### Xenograft Radio-Sensitivity Assay

When the xenograft tumors reached to an average volume of 150 mm^3^, 10 Gy irradiation was delivered using a linear accelerator (23EX; Varian, United States) as previously described (Peng et al., 2018). The growth of the xenografts was evaluated every 3 days until the 18th day of irradiation.

### Statistical Analysis

Data were analyzed using SPSS 23.0 (SPSS Inc, Chicago, IL, United States). Data with normal distribution were represented by the mean ± SD and analyzed by an independent *t* test. The data with a non-normal distribution were represented by median (interquartile range (IQR), P_25_–P_75_) and analyzed by the Mann–Whitney *U* test or Kruskal–Wallis H test. Unconditional logistic regression was used to estimate the odds ratio (OR) of ESCC with 95% confidence intervals (CI) for BCLs, and the chi squared test for trend was calculated. Univariate and multivariate models were used to analyze clinicopathological parameters for correlation with OR of ESCC. Kaplan–Meier log-rank tests were used to analyze progression-free survival (PFS) by various factors, and then modeled by multivariable Cox proportional-hazard regression models, estimating hazard ratios (HRs) and 95% CIs. All tests were two-sided and a *p* < 0.05 was considered statistically significant.

## Results

### BCLs Are Associated With ESCC Risk and Prognosis

**FIGURE 1:**
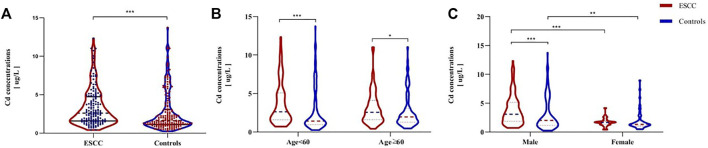
Comparison of BCLs between ESCC patients and healthy controls **(A)** and BCLs in cases and controls according to their age and sex **(B–C)**. *p*-values were calculated using the Mann–Whitney *U* test. *p < 0.05; **p < 0.01; ***p < 0.001, compared with the designated controls.

The BCLs in the patients and controls according to gender and age were shown in Table 1. The median concentration of BCL is higher in the ESCC patients (2.60, 1.60–4.71 μg/L) than in the controls (1.63,1.14–3.07 μg/L) (*p* < 0.001; Figure 1A), regardless of age (Figure 1B). BCLs were associated with increased risk of ESCC, based on an OR of 1.11 (95% CI 1.01, 1.21), and there was a dose-response relationship between the BCLs and ESCC risk by the trend test (*p* < 0.001), with the ORs of 3.12 (95% CI 1.54, 6.30) and 3.71 (95% CI 1.84, 7.48) in the third and fourth quartiles, respectively. In addition, male patients tended to have higher BCLs than the male controls, while there was no difference found in the female participants (*p* < 0.001, Figure 1C).

**TABLE 1:** BCLs in the survey population and the associations between ESCC risk and BCLs.

Groups	Controls		Patients		Or (95% CI)	*p*
	n	BCLs (μg/L), median (P25, P75)	n	BCLs (μg/L), median (P25, P75)		
Total	177	1.63 (1.14,3.07)	150	2.60 (1.60,4.71)	1.11 (1.01,1.21)	0.04^a^
<1.14	45	0.84 (0.57,0.96)	16	0.92 (0.72,1.06)	Reference	
1.14–1.63	44	1.31 (1.22,1.46)	23	1.43 (1.30,1.49)	1.49 (0.70,3.21)	0.30^a^
1.63–3.07	44	2.21 (1.95,2.43)	50	2.26 (1.82,2.64)	3.12 (1.54,6,30)	<0.01^a^
>3.07	44	6.28 (4.95,7.51)	61	5.08 (3.97,7.01)	3.71 (1.84,7.48)	<0.001^a^
Trend Gender						<0.001^a^,^b^
Female	51	1.33 (1.09,1.85)	31	1.66 (1.32,1.82)	Reference 1.55 (0.93,2.59)	0.09
Male	126	2.01 (6.11,8.40)	119	3.08 (1.87,5.11)		
Age						
<60	94	1.42 (0.98,3.46)	41	2.62 (1.61,5.02)	Reference	
≥60	83	1.95 (1.28,3.02)	67	2.57 (1.60,3.98)	1.26 (0.82,1.95)	0.30

Blood cadmium levels (BCLs), Odds ratios (OR), 95% confidence intervals (CI), and *p* < 0.05 was considered statistically significant.

a Adjusted for age and gender.

b Analyzed by chi square test for trend.

Using the median concentration of 2.61 μg/L in the controls as the threshold value of BCLs, the associations between clinicopathological features and subjects by Cd burden status (i.e., <2.61 vs. ≥2.61 μg/L) was evaluated by univariate and multivariate logistic regression (Table 2). In the univariate model analysis, male patients (*p* < 0.001), cases with a smoking history (*p* < 0.001) or alcohol consumption (*p* < 0.001), cases with no disease history (*p* = 0.05) or with low body mass index (BMI) (*p* = 0.02) tended to have more Cd accumulation. However, further multivariate logistic regression analysis excluded the role of tobacco (*p* = 0.34) or alcohol (*p* = 0.11) in BCLs.

**TABLE 2:** Association between clinicopathological characteristics and BCLs in ESCC patients.

Variables	BCLs		Univariate model		Multivariate model	
	<2.60 µg/L, *n* = 75	≥2.60 µg/L, *n* = 75	OR (95% CI)	*p*	OR(95% CI)	*p*
Gendar						
Female	28 (37.33)	3 (4.00)	1		1	
Male	47 (62.67)	72 (96.00)	14.30 (4.11, 49.71)	<0.001	16.86 (3.11, 91.58)	0.001
Age						
<60	35 (46.67)	36 (48.00)	1	0.87	1	0.13
≥60	40 (53.33)	39 (52.00)	0.95 (0.50, 1.80)		2.01 (0.81, 4.98)	
Smoking history						
NO	44 (58.67)	15 (20.00)	1	<0.001	1	0.34
YES	31 (41.33)	60 (80.00)	5.68 (2.74, 11.77)		1.66 (0.58, 4.75)	
Alcohol consumption						
NO	64 (85.33)	41 (54.67)	1	<0.001	1	0.11
YES	11 (14.67)	34 (45.33)	4.83 (2.20, 10.58)		2.34 (0.83, 6.61)	
Disease history						
NO	47 (62.67)	58 (77.33)	1	0.05	1	0.01
YES	28 (37.33)	17 (22.67)	0.49 (0.24, 1.00)		0.27 (0.10, 0.74)	
Family history of cancer						
NO	71 (94.67)	70 (93.33)	1	0.73	1	0.76
YES	4 (5.33)	5 (6.67)	1.27 (0.33, 4.92)		1.31 (0.23, 7.40)	
Clinical stages						
I	2 (2.67)	4 (5.33)	1	0.2	1	0.13
II	20 (26.67)	12 (16.00)	0.3 (0.05, 1.89)	0.61	0.07 (0.02, 2.24)	0.40
III	34 (45.33)	43 (57.33)	0.63 (0.11, 3.66)	0.35	0.19 (0.04, 8.57)	0.12
IV	19 (25.33)	16 (21.33)	0.42 (0.07, 2.61)		0.03 (0.00, 2.45)	
N classification						
N0	20 (26.67)	18 (24.00)	1	0.50	1	0.47
N1	33 (44.00)	39 (52.00)	1.31 (0.60, 2.89)	0.62	0.59 (0.14, 2.49)	0.70
N2	21 (28.00)	15 (20.00)	0.79 (0.32, 1.99)	0.32	0.70 (0.11, 4.41)	0.36
N3	1 (1.33)	3 (4.00)	3.33 (0.32, 34.99)		4.78 (0.17, 138.44)	
T classification						
T1	6 (8.00)	5 (6.67)	1	0.54	1	0.39
T2	5 (6.67)	7 (9.33)	1.68 (0.32, 8.76)	0.85	3.63 (0.19, 69.01)	0.96
T3	34 (45.33)	25 (33.33)	0.88 (0.24, 3.22)	0.52	1.07 (0.09, 13.01)	0.72
T4	30 (40.00)	38 (67)	1.52 (0.42, 5.47)		1.62 (0.12, 21.63)	
M classification						
M0	66 (88.00)	65 (86.67)	1	0.81	1	0.27
M1	9 (12.00)	10 (13.33)	1.13 (0.43, 2.96)		2.82 (0.45, 17.56)	
BMI (continuous)	21.72 ± 3.84	20.36 ± 2.74	0.87 (0.77, 0.98)	0.02	0.77 (0.64, 0.93)	0.01

Odds ratios (OR), 95% confidence intervals (CI), and *p* ± 0.05 was considered statistically significant; T classification, size of the primary tumor; N classification, lesion of regional lymph nodes; BMI, body mass index.

Data are no. (%) unless indicated.

**FIGURE 2:**
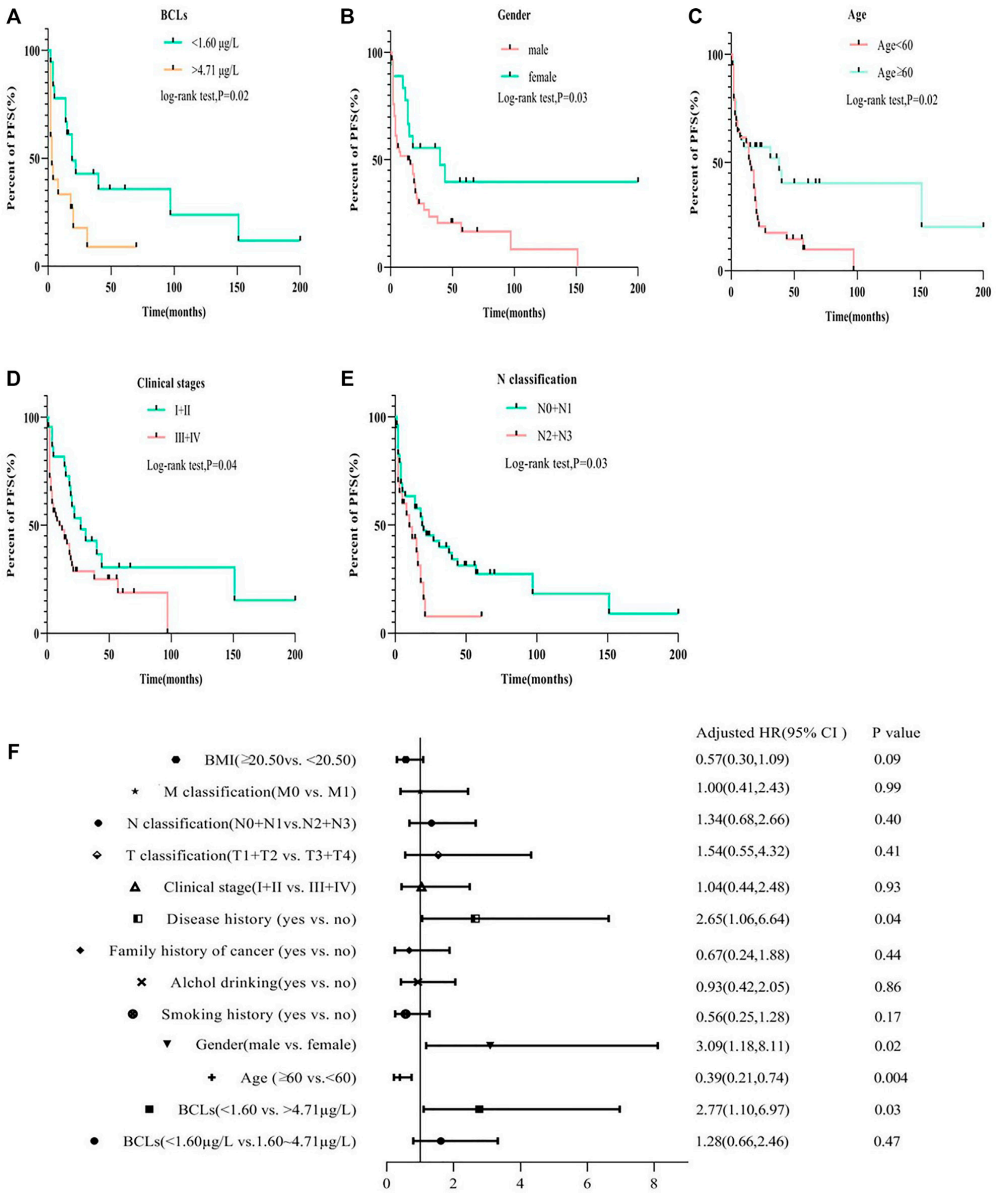
Compare the PFS of ESCC patients with Cd burden, gender, age, clinical stage, and N classification by Kaplan–Meier log-rank tests **(A–E)** and multivariate Cox analysis for the PFS risk related to different groups **(F)**.

The results of Kaplan-Meier analysis showed that BCL, gender, age, clinical stage, and N classification were all associated with PFS (log-rank test; all *p* < 0.05; Figures 2A–E). PFS decreased with increasing clinical stage and N classification (*p* = 0.04; *p* = 0.03). Specifically, patients being male and young at diagnosis had a shorter PFS (*p* = 0.03; *p* = 0.02). With reference to quartile analysis, patients with the highest BCL quartile have a shorter PFS than that of the lowest quartile (>4.71 μg/L vs. <1.6 μg/L,log-rank test,*p* = 0.04), No association was found in smoking or alcohol history, family history of cancer, disease history, T or M classification related to PFS (log-rank test; all *p* > 0.05; Supplementary Figure S1). The Cox proportional hazard regression model revealed that in addition to gender (HR = 3.09; 95% CI 1.18–8.11; *p* = 0.02), disease history (HR = 2.65; 95% CI 1.06–6.64; *p* = 0.004), age at diagnosis (HR = 0.39; 95% CI 0.21–0.74; *p* = 0.004), BCL (<1.60 vs. > 4.71 μg/L) (HR = 2.77; 95% CI 1.10–6.97; *p* = 0.03) was an independent prognostic factor for ESCC progression (Figure 2F).

### Chronic Cd Exposure Promotes Proliferative and Metastatic Phenotypes of ESCC Cells Both *in Vitro* and *in Vivo*

**FIGURE 3:**
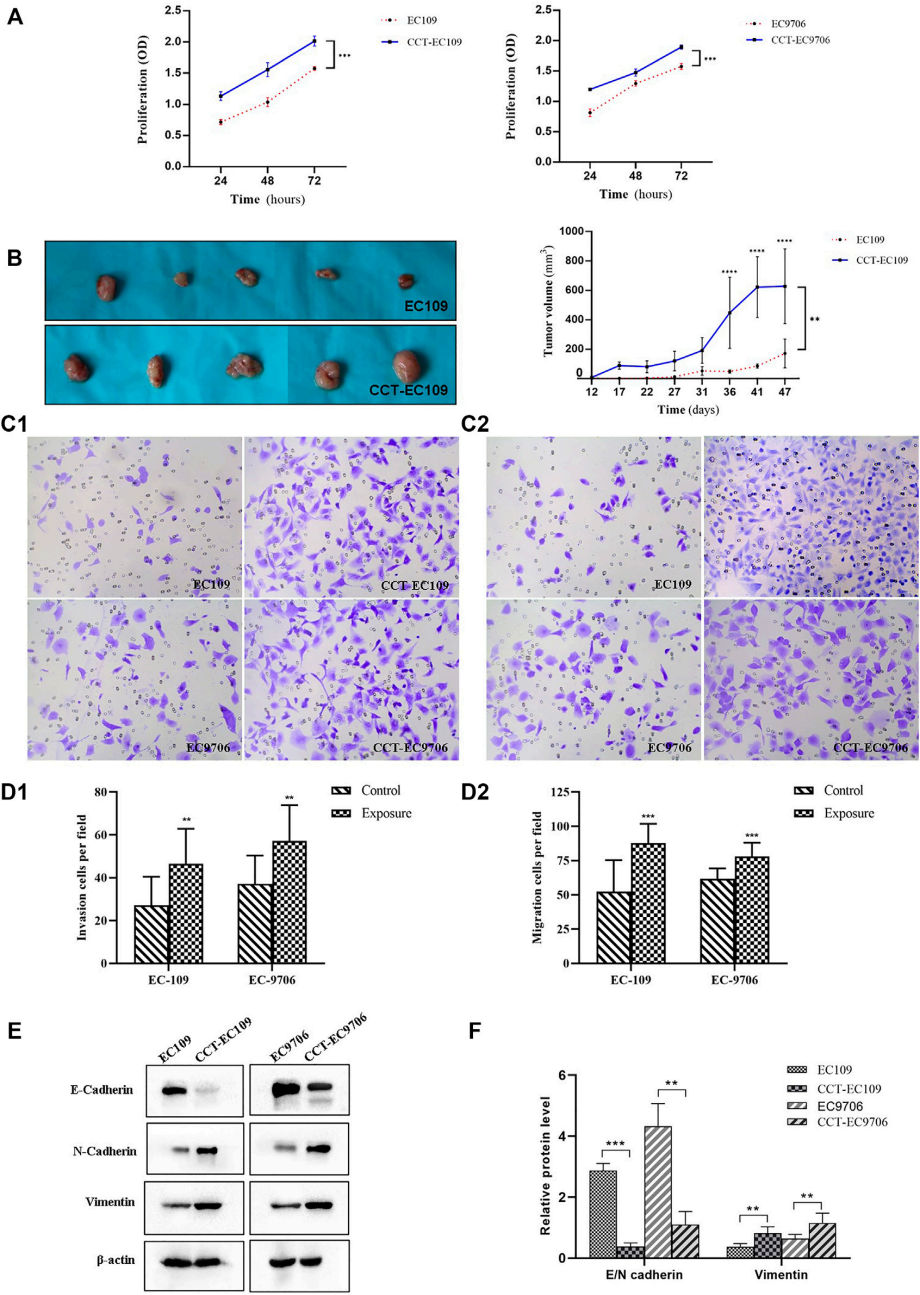
Cell proliferation was determined by MTT assay in EC109 (left panel) and EC9706 (right panel) cells following 5 μM Cd treatment for 10 weeks **(A)**. Comparison xenograft tumor growth between EC109 and CCT-EC109 xenograft mice (n = 5) **(B)**. Invasion was determined by transwell assays with Matrigel (magnification ×100) **(C1,D1)** and migration was assessed by transwell assay without Matrigel **(C2,D2)**. Representative Western blot image and the corresponding quantitative analyses for the expression of EMT biomarkers (E-cadherin, N-cadherin and vimentin in ESCC cells with the presence and absence of Cd **(E,F)**. The values are mean ± SD from three independent experiments. Results are presented as mean ± SD from three independent experiments. *p < 0.05; **p < 0.01; ***p < 0.001, compared with the controls.

Continuous exposure of EC109 and EC9706 cells to 5 μM CdCl_2_ for 12 weeks resulted in a marked increase in cell proliferation compared with the parental cells (Figure 3A, *p* < 0.001). Cd-stimulated tumorigenicity was also demonstrated by xenograft tumor experiments in which the nude mice injected with CCT-EC109 exhibited more rapid formation of tumors compared to that of EC109 cells, especially in the period from day 36 post injection (Figure 3B, *p* < 0.01).

Transwell assays were performed to assess cell migration and invasion. The results showed that the invasive capacity of CCT-EC109 and CCT-EC9706 cells was markedly increased with 1.72- (*p* < 0.01) and 1.54-(*p* < 0.01) fold of their parental controls, respectively (Figure 3C). Similarly, CCT-EC109 and CCT-EC9706 cells displayed robust migration as compared with the controls, as the number of transmigrated cells was 1.68 and 1.26 times greater than that of EC109 (*p* < 0.001) and EC9706 (*p* < 0.01) cells, respectively (Figure 3D). And the ratio of invasive cell number/migratory cell number Moreover, we observed the hallmark of EMT, E-cadherin/N-cadherin switch and vimentin upregulation in the CCT-EC109 and CCT-EC9706 cells (Figures 3E,F).

### Chronic Cd Exposure Decreases Cellular Response to Chemotherapeutic Agents and Irradiation in Cd-Treated Cells

**FIGURE 4:**
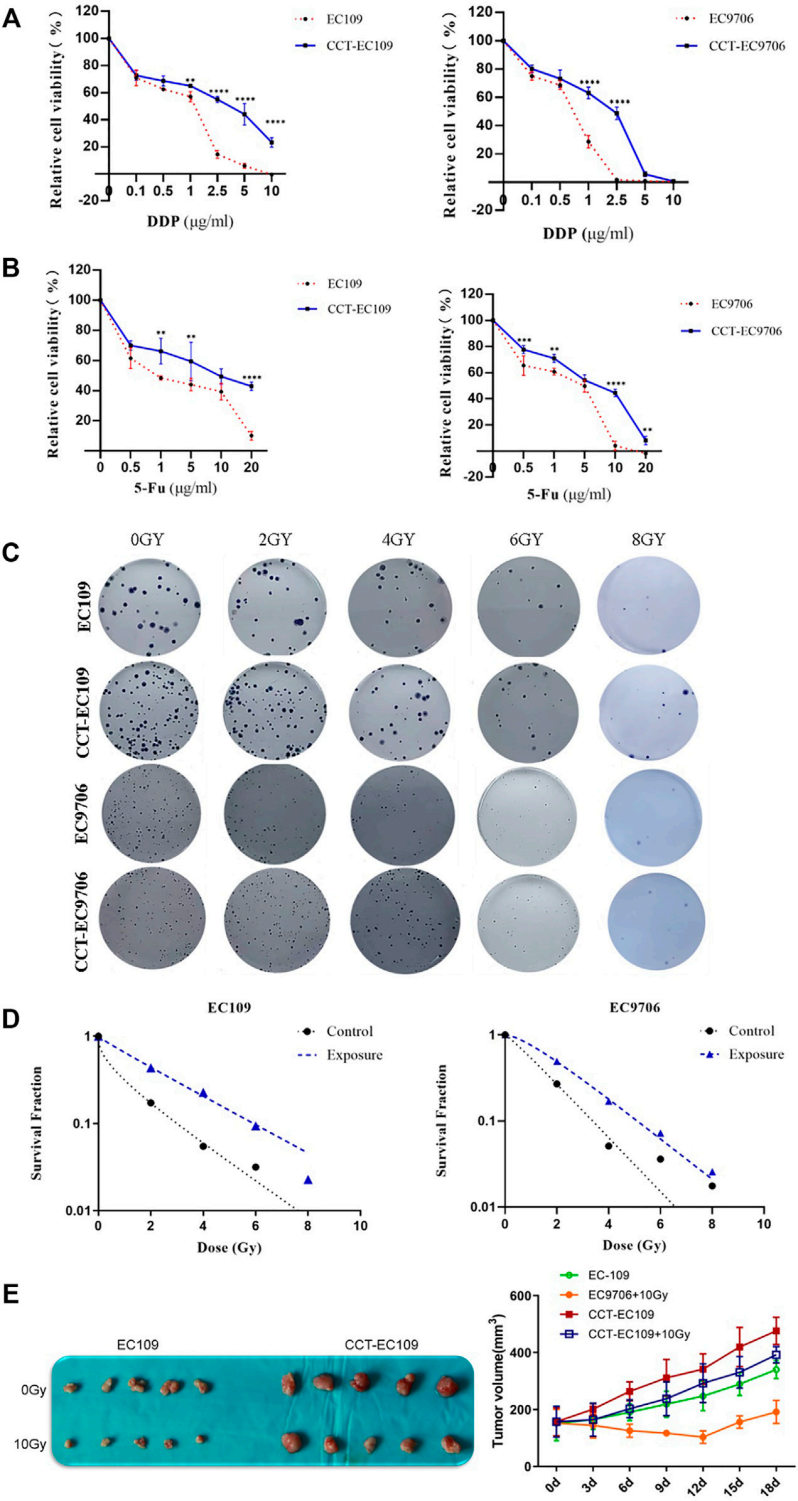
MTT assay on the relative cell viability of CCT-EC109 and CCT-EC9706 and their parental cells before and after treatment with DDP **(A)** or 5-FU **(B)** for 48 h. Representative colony formation for different cell groups following irradiation **(C)** and survival curves for different cell groups following irradiation at different doses were fitted according to the multi-target single-hit model **(D)**. Representative photos (left panel of **(E)** and growth curves (right panel of E) for xenograft tumors following injection with EC109 and CCT-EC109 cells combined with 10Gy irradiation. Data represent mean ± SD of three independent experiments. *p < 0.05; **p < 0.01; ***p < 0.001.

MTT assays were performed to determine the chemosensitivity of EC109 and EC9706 cells with or without chronic Cd exposure. As depicted in Figure 4A, CCT-EC109 and CCT-EC9706 cells exhibited attenuated sensitivity to DDP. Similarly, the 2 cell lines exhibit tolerance to 5-FU as compared with their parental cells (Figure 4B).

Radiation resistance induced by Cd was explored both *in vitro* and *in vivo*. Following irradiation with different doses of X-rays (0, 2, 4, 6 and 8 Gy), higher SF value was observed in the CCT-EC109 and CCT-EC9706 cells compared with the controls (Figure 4C). The SER values for the Cd treated groups were 0.043 in CCT-EC109 cells and 0.624 for CCT-EC9706 cells, respectively (Figure 4D). Moreover, CCT-EC109 xenograft tumors grew slower than that of EC109 group after 10 Gy irradiation (Figure 4E). In addition, we used two Cd-treated nasopharyngeal carcinoma (CCT-NPC) cell lines CCT-CNE1 and CCT-CNE2, that had been continually exposed to 1 μM CdCl2 for 14 weeks in our previous study (Peng et al., 2019), to further confirm the universal radioresistance effect of Cd. Consistently, the numbers of surviving clones in the CCT-CNE1 and CCT-CNE2 cells following 2 and 4Gy irradiation were significantly higher than that of parental cells (Supplementary Figure S2).

### Prolonged Cd Exposure Induces CSC Characteristics in ESCC Cells

**FIGURE 5:**
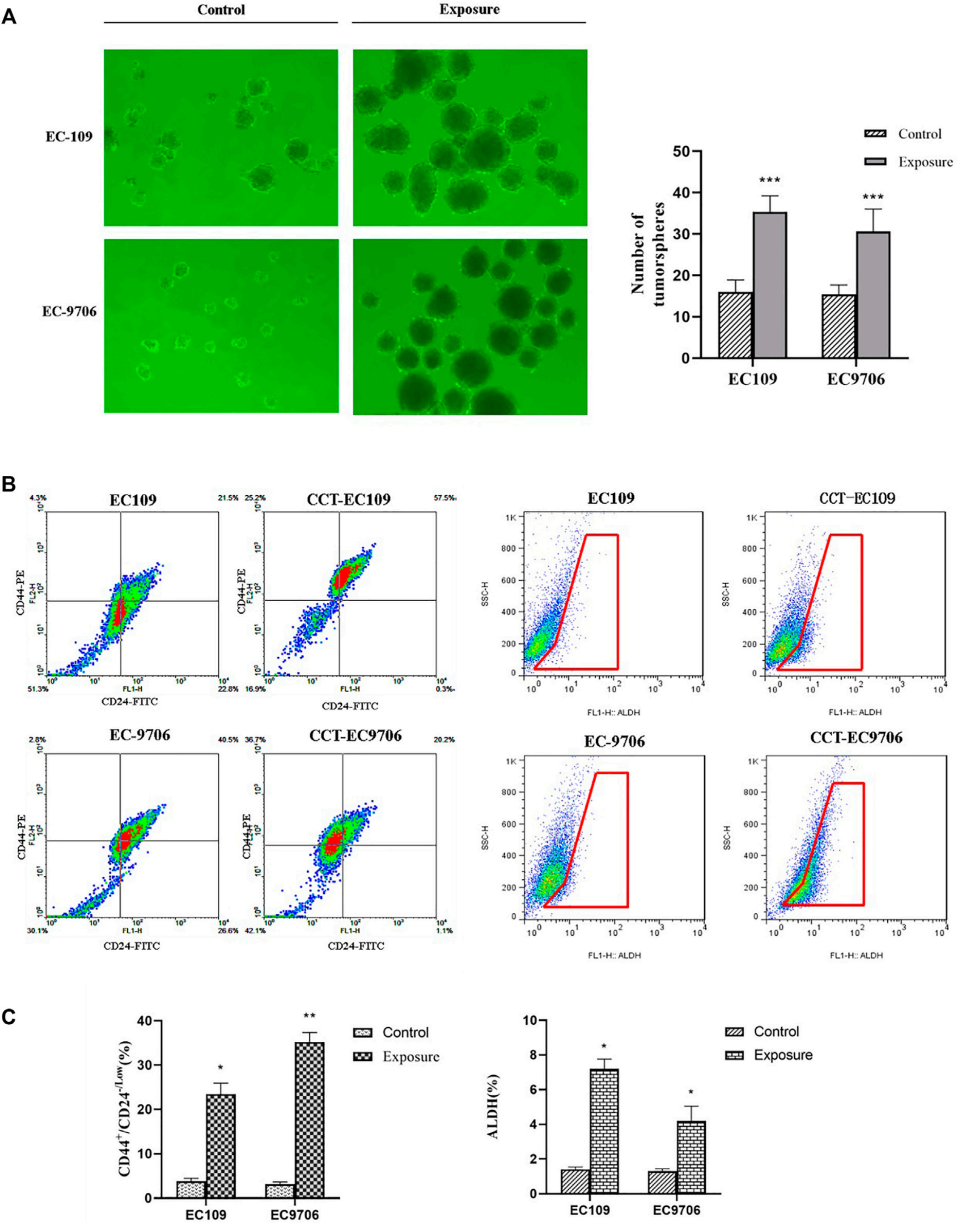
Number of tumor spheres over 50 cells was counted in the Cd exposed and control groups **(A)** and flow cytometric analysis for CD44^+^/CD24^-/Low^ (left panel of **(B,C)** and ALDH (right panel of B&C) expression in CCT-ESCC cells and controls. Data present Mean ± SD from 3 independent experiments. *p < 0.05, **p < 0.01, ***p < 0.001.

Emerging evidence on metal carcinogenicity has suggested the induction of CSC-like property was involved in the mechanism of Cd carcinogenesis (Wang and Yang, 2019). Therefore, we explored the impact of Cd on the formation of tumor sphere. The molecular biomarkers that have been used to identify and isolate cell populations with CSC properties, such as CD44^+^/CD24^-^ and aldehyde dehydrogenase (ALDH) (Toledo-Guzmán et al., 2019), were examined in the CCT-ESCC cells as well as the parental cells. Tumor sphere formation assay showed that the tumor spheres formed from the CCT-EC109 and CCT-EC9706 cultures were more numerous and larger than that from EC109 (0.338 ± 0.036 vs. 0.080 ± 0.015, *p* < 0.001) and EC9706 (0.208 ± 0.031 vs. 0.077 ± 0.012, *p* < 0.001) cells (Figure 5A). The percentages of CD44^+^/CD24^-^ cells in CCT-EC109 and CCT-EC9706 cells were 6.17- and 11.17-fold higher, respectively, than the controls (Figures 5B,C, left panel). Similarly, both CCT-EC109 and CCT-EC9706 cells contained a larger fraction of ALDH^+^ cells, accounting for 7.2 ± 0.57% and 4.2 ± 0.85% of the population, respectively, as compared to the expression of ALDH^+^ cells in EC109 and EC9706 with 1.4 ± 0.14% and 1.3 ± 0.14%, respectively (Figures 5B,C, right panel). These findings indicated that chronic Cd increases the CSC phenotype.

### Epigenetic Regulation of Wnt/β-Catenin Involves in Cd Carcinogenesis and Therapeutic Resistance in ESCC Cells

**FIGURE 6:**
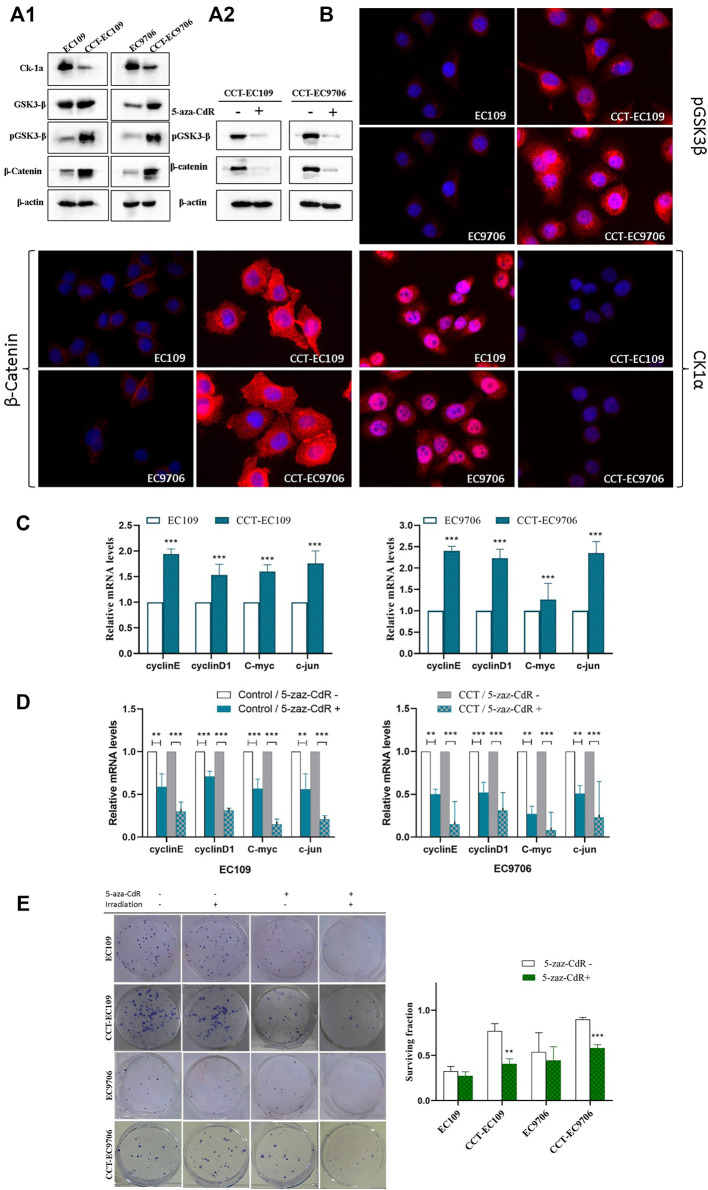
Western blot analysis on the expression of CK1α, GSK3β, p-GSK3β and β-catenin in CCT-ESCC and their parental cells **(A1)**, and with or without 5-aza-CdR treatment in CCT-ESCC cells **(A2)**. and immunofluorescence staining of p-GSK3β, β-catenin and CK1α in the exposed and control cells (40×) **(B)**. qRT-PCR results for relative transcript levels of Wnt signaling pathway target genes in CCT-ESCC cells and control cells **(C)**. and with or without 5-aza-CdR treatment in CCT-ESCC cells and the controls **(D)**. Gross view of colony formation (left panel) and corresponding quantitative analyses of the relative surviving fraction in CCT-ESCC cells and control cells **(E)**. β-actin was used as the housekeeping gene and raw data was analyzed by the 2^−ΔΔCt^ method. The surviving fraction was calculated as follows (number of colonies/number of cells plated)_irradiated_/(number of colonies/number of cells plated)_nonirradiated_. Data represent mean ± SD of three independent experiments. *p < 0.05; **p < 0.01; ***p < 0.001.

Upregulation of β-catenin and p-GSK3β, as well as decreased expression of CK1α, was observed in both CCT-EC109 and CCT-EC9706 cells, as demonstrated by Western blotting (Figure 6A1) and immunofluorescence analyses (Figure 6B). Subsequent quantitative qRT–PCR analysis results confirmed the elevated transcription of target genes downstream of Wnt signaling pathway, including *c-Myc, c-jun, cyclin D1 and cyclin E*, following chronic Cd exposure (Figure 6C). These results suggest that prolonged Cd exposure elevated the activity of the canonical Wnt signaling pathway in CCT-ESCC cells.

Since epigenetic deregulation of Wnt/β-catenin, especially promoter methylation of tumor suppressor genes, has been implicated in the molecular pathology of ESCC (Singh et al., 2019), as well as in CSC fate modulation (Deshmukh et al., 2017), we treated CCT-ESCC cells with the demethylating agent 5-aza-CdR to explore the role of DNA methylation in this CCT-ESCC model. The results, as shown in Figure 6A2, indicated that 5-aza-CdR was able to downregulate the expression of p-GSK3β and β-catenin (Figure 6A2) in both CCT-EC109 and CCT-EC9706 cells. Further analysis showed that demethylation repressed the transcription of target genes of β-catenin signaling in both the exposed cells and their parentals (Figure 6D), but the alterations in exposed group showed more obviously. Importantly, 5-aza-CdR treatment significantly rescued the radioresistance in both CCT-EC109 and CCT-EC9706 cells while slight enhancement of radiosensitivity could be found in their parental cells with no significant differences (Figure 6E).

## Discussion

The present study indicates a significant association between elevated risk of ESCC and blood Cd burden among individuals without occupational exposure particularly in men and subjects with no disease history and with lower BMI. Although the median BCL in ESCC female patients was higher than that of controls, no significant difference could be found. The possible reason may be due to the small size of the sample. These observations support the etiological link between environmental Cd exposure and esophageal cancer. A prior hospital-based study from Pakistan with 21 male esophagus cancer patients and controls, demonstrated that in addition to the lower levels of Zn and Se found in the scalp hair samples, the mean concentrations of arsenic, nickel and Cd were significantly higher in the cases as compared to the controls (Kazi et al., 2015). Some evidence from environmentally disadvantaged regions indicated the correlation between high levels of Cd in drinking water and the high prevalence of esophageal cancer (Amer et al., 1990; Mamyrbayev et al., 2016). Nevertheless, a disease-mapping study from Norwich, a city that was exposed to zinc Cd sulfide during the cold war reported conflicting data suggesting environmental pollution unlikely influence esophageal cancer risk (Beale et al., 2010). The current study results were consistent with our earlier study displaying Cd and lead exposure increased the gastrointestinal cancer risk in the Chaoshan population. It showed the median BCL in gastrointestinal cancer patients was significantly higher than that of controls (2.12 vs. 1.47 μg/L, *p* < 0.001) and individuals with BCLs>2.12 μg/L had higher risk of gastrointestinal cancers (Lytle et al., 2018). These findings indicated that assessment of the Cd burden in the general population might help early identification of subjects with potential of high risk for ESCC. Moreover, our results with reference to quartile analyses indicate that a significant association between BCLs and ESCC risk might exist at high Cd exposure levels, suggesting that Cd exposure above the threshold might become a concern.

In the current study, multivariate logistic regression analysis revealed that high BCLs in patients were associated with gender, disease history and BMI, while alcohol or tobacco consumption had no role in blood Cd burden. These results accord with these earlier findings that tobacco and alcohol consumption were not significant factors in any of the major epidemic ESCC rural populations of the world whereas environmental and nutritional factors were accounted (Freedman et al., 2007; Kmet and Mahboubi, 1972; Rensburg, 2019; Tran et al., 2005). Also, the strong correlation between BCLs and male gender in current study is consistent with the gender difference with 2 to 3-fold increase in incidence and mortality of ESCC in males according to Global cancer statistics 2020 (Sung et al., 2021).

A previous study indicated that the mortality from all cancers and esophageal cancer in a Chinese population living around a multi-metal sulphide mine were elevated compared with the mortality in a low-exposure area (Wang et al., 2011). In the present study, no significant association was found between BCLs and overall survival (OS) in these ESCC patients. It could be argued that the negative results were due to the much lower BCLs in our survey than the geometric mean of BCL (24.10 μg/L) in the contaminated population. Notably, with reference to quartile analysis, patients with the highest BCL quartile have a shorter PFS than that of lowest quartile, suggesting that BCL may serve as a potential independent indicator for poor prognosis of ESCC patients.

Recently, an experimental study suggested that CDK6 upregulation resulting in inhibition of human esophageal epithelial cell apoptosis might involve in the etiological mechanism of Cd-induced esophageal cancer (Yang et al., 2020). However, it is worth noting that the exposure model used was based on short-term Cd exposure below 24 h and therefore no malignant transformation could be observed. It has been indicated that prolonged Cd treatment in bronchial, lung, prostate or breast epithelial cells induced malignant transformation with hyperproliferation and increased potential to invade and migrate (Achanzar et al., 2001; Benbrahim-Tallaa et al., 2009; Person et al., 2013). Similarly, exposure of breast cancer cells and nasopharyngeal carcinoma cells to Cd promoted cell malignant phenotypes (Achanzar et al., 2001; Benbrahim-Tallaa et al., 2009; Peng et al., 2019; Person et al., 2013; Wei and Shaikh, 2017). Consistent with the literature, the present research also observed chronic Cd exposure conferred ESCC cells a growth advantage and metastasis-associated phenotype, as evidenced by enhanced proliferation, invasion and migration. These results established a cause-and-effect association between chronic low-level Cd exposure and ESCC progression, showing Cd not only conferred ESCC cells a growth advantage *in vitro* and *in vivo*, but also stimulated metastasis-associated phenotype, as evidenced by enhanced invasion and migration along with EMT, characterized by cadherin switch. These results corroborate the findings of previous work in neoplastic transformation of non-cancer epithelial cells induced by Cd, which showed chronic Cd exposure induced epithelial cells into a more invasive and migratory phenotype by inducing EMT (Vanlaeys et al., 2020). EMT program has been shown to be crucial in tumor initiation, metastasis, cancer stemness and resistance to chemotherapy and radiation, and been associated with inferior cancer survival rate as well (Cheung et al., 2011; Loh et al., 2019; Roy et al., 2021). Combined with our earlier observations in chronic Cd-treated nasopharyngeal carcinoma cell model (Peng et al., 2019), these findings strongly suggest the carcinogenic and tumor-promoting effect of chronic Cd exposure in various human cancers.

Precision medicine is an emerging approach taking into account of individual variability in genes, environment, and lifestyle factors (Saadeh et al., 2019). A previous investigation on possible connection between Cd and chemosensitivity was conducted in breast cancer cells exposed to Cd for several days, which demonstrating that 5-FU cytotoxicity on the MCF-7 breast cancer cell line could be reduced by Cd treatment for 24 h (Asara et al., 2012). The present research suggests that long-term Cd treatment not only prevents the cytotoxic effect of 5-FU and DDP on CCT-EC109 and CCT-EC9706 cells, but also enhances the radiation resistance in both CCT-ESCC cells and CCT-CNE1 and CCT-CNE2 cells as well. To the best of our knowledge, this is the first study to demonstrate Cd exposure confers radio-resistance in cancer cells. These results suggest that Cd exposure is likely to be related to chemoradioresistance, which may help us understand the role of environmental factors in inter-individual variability in responses to antineoplastic drugs and radiation. Generally, acquisition of chemo- or radio-resistance ultimately results in esophageal cancer relapse and therefore poor prognosis. Thus, these findings may explainprevious reports that ESCC patients exposed to long-term environmental Cd had an increased risk of mortality (Wang et al., 2011). It may also support the observation of shorter PFS for ESCC patients with high Cd burden in the present human study.

The cancer cell stem-like properties were observed in CCT-ESCC cells, as evidenced by the tumor sphere formation and increased expression of CSC biomarkers CD44 and ALDH. Environmental pollutants have been suggested to be one of the causative factors for the generation of the CSCs. Our findings support previous observations in other Cd-exposure model systems representing multiple organ types. For instance, elevated CSC populations and increased mRNA expression of CD44 and ALDH1 have been shown in MCF-7 and HepG2 cells in response to the treatment of 0.1–1.0 mM Cd for 72 h (Ju et al., 2017). Also, human HPDE pancreatic epithelial cells exposed to chronic Cd treatment displayed acquisition of multiple characteristics typical of CSCs (Qu et al., 2012). Activation of stem cells in cancers has been suggested to lead to progression, therapy resistance and metastatic growth (Lytle et al., 2018). The current finding suggests the biological plausibility of a therapy resistance-establishment of stemness relation concerned with Cd carcinogenic effects in ESCC. Nevertheless, to fully explore the stemness induced by Cd in ESCC initiation and development, further studies with more focus on Cd orchestrating CSC-like properties in esophageal epithelial cells is suggested.

The effects of Cd on cancer stem cell-related signaling pathways have been addressed in Cd treated-MCF-7 and HepG2 cells, i.e. the activation of Ras/Raf/MEK/ERK signaling cascade (Ju et al., 2017) and the activation of MAPK/ERK signaling pathway in human lung adenocarcinoma cells (Huff et al., 2016). It is clear that hyperactivated Wnt signaling plays critical role in the pathogenesis of esophageal cancer, cancer progression and chemo- and radio-resistance (Su et al., 2016; Wei et al., 2021). Our earlier observations in prolonged Cd-treated nasopharyngeal carcinoma cells showed that chronic Cd exposure aggravates malignant phenotypes of nasopharyngeal carcinoma by activating the Wnt/β-catenin signaling pathway via hypermethylation of the casein kinase 1α promoter (Peng et al., 2019). Similarly, the present research revealed Cd exposure induced aberrant activation of Wnt/β-catenin pathway in ESCC cells. Prior studies that have noted treatment of esophageal cancer cells with 5-aza-CdR could suppress the Wnt/β-catenin signaling activation (Sun et al., 2021) and resensitize cells to irradiation (Sakakura et al., 2007). In present, one interesting finding is demethylation was able to downregulate the activity of Wnt/β-catenin pathway and reverse the radioresistance in both exposed cells and their parentals, nevertheless, the rescue effect on CCT-ESCC model exhibited more obviously. Collectively, this study suggests that the epigenetic activation of canonical Wnt signaling pathway is involved in Cd carcinogenesis and Cd-associated therapeutic resistance in ESCC.

The present work provides both epidemiological and experimental evidence for the connection between chronic Cd exposure and ESCC development and therapeutic resistance in ESCC. Also, we identified an increase in cancer stemness and activation of canonical Wnt signaling pathway linked with DNA methylation playing roles in the carcinogenic potential of Cd in ESCC.

There are some limitations in this study. Firstly, we used blood Cd concentrations but not urinary Cd to estimate the heavy metal burden in participants. It is generally believed that dietary intake is one of the major environmental sources of Cd exposure in the general population (Hartwig, 2013). A literature-based survey on population with no occupational exposure indicates that Cd levels in urine and blood are related to diet Cd intake with the correlation coefficients of 0.570 (*p* < 0.01) and 0.792 (*p* < 0.01), respectively. This study suggests the possibility of estimating dietary Cd burden from Cd in blood for the general population (Ikeda et al., 2011). In another recent case-control study, BCL was proposed as a valuable indicator for early lung cancer detection (Lener et al., 2021). Furthermore, the identification for environmental sources or dietary exposure to Cd were not included in this investigation, hence we were not able to adjust for other cofounders which could determine Cd exposure. Thirdly, having in mind that other trace elements especially heavy metals can contribute to ESCC susceptibility, progression and treatment resistance, evaluation of trace elements profile in subjects and the complex interaction among trace elements might help to better understand the implication of Cd in ESCC progression. Lastly, the present study focuses on cancer stemness and the Wnt signaling pathway involved in the mechanism of Cd-induced ESCC progression and chemoradioresistance. Further studies are needed to clarify whether these mechanisms involved in Cd-induced malignant transformation in normal esophageal epithelial cells.

## Conclusion

The current work elucidates the association between chronic Cd exposure and the development and progression of ESCC. Additionally, our study expands the body of knowledge concerning Cd exposure in cancer chemo- and radio-resistance. Our study suggests that screening for body Cd burden by monitoring BCLs could be used to identify patients with high-risk of ESCC, and to predict outcomes prior to treatment. In clinical practice, monitoring the body Cd level for evaluation of ESCC patients warrants further research.

## Data Availability

The raw data supporting the conclusions of this article will be made available by the authors, without undue reservation.

## Abbreviations

ALDH, aldehyde dehydrogenase; BCL, blood cadmium level; CSC, cancer stem cell; DDP, cisplatin; EMT, epithelial–mesenchymal transition; ESCC, esophageal squamous cell carcinoma; 5-Fu, 5-Fluorouracil; MTT, 3-(4,5-dimethylthiazol-2yl)-2,5-diphenyltetrazolium bromide; PFS, progression-free survival.
