# Supplementary figures and images for: Environmental Cadmium Exposure Promotes the Development, Progression and Chemoradioresistance of Esophageal Squamous Cell Carcinoma
Source: Front Cell Dev Biol. 2022 Feb 18;10:792933. doi: 10.3389/fcell.2022.792933 (PMC8894704; doi:10.3389/fcell.2022.792933)

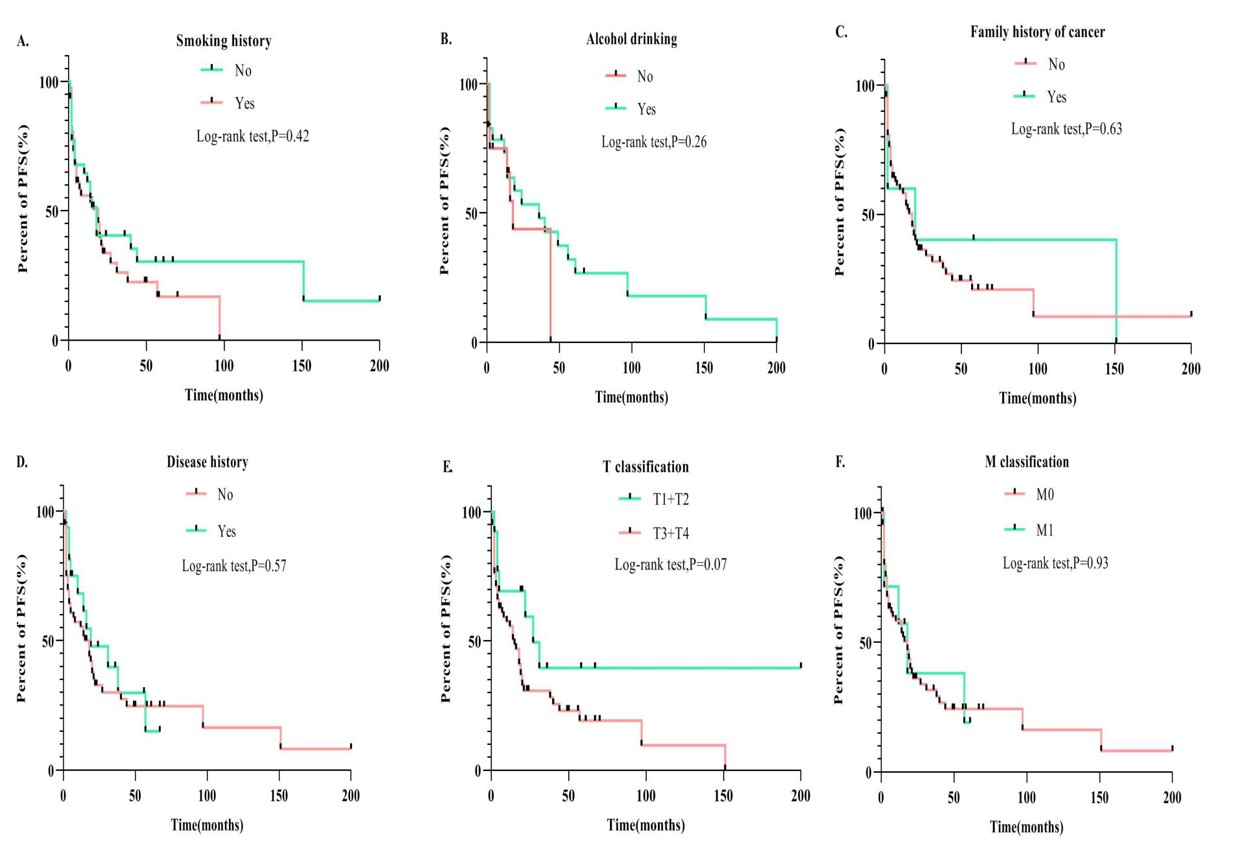

Supplement: Supplementary file 1 [file Image3.JPEG]

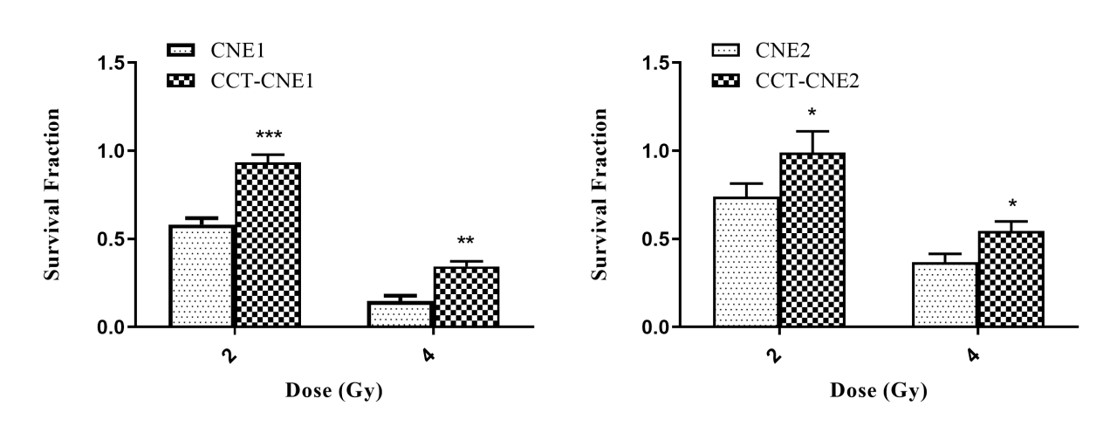

Supplement: Supplementary file 2 [file Image4.JPEG]

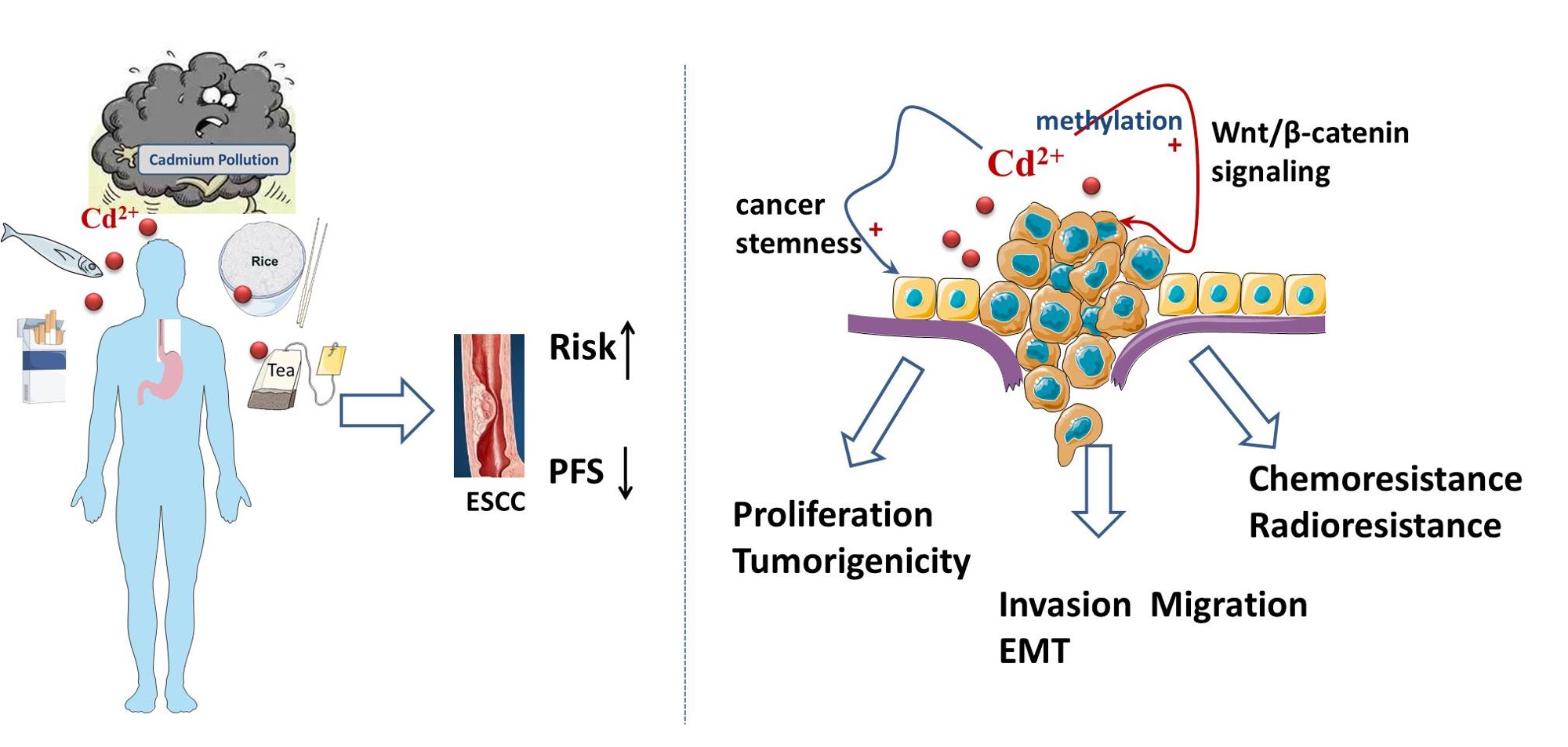

Supplement: Supplementary file 3 [file Image2.JPEG]
