# Supplementary material for: Environmental Cadmium Exposure Promotes the Development, Progression and Chemoradioresistance of Esophageal Squamous Cell Carcinoma
Source: Front Cell Dev Biol. 2022 Feb 18;10:792933. doi: 10.3389/fcell.2022.792933 (PMC8894704; doi:10.3389/fcell.2022.792933)
Supplement: Supplementary file 4 [file Table3.DOCX]

**Table S1 Demographic characteristics of the participants**

|  | Cases(n=150) | Controls(n=177) | *P* |
| --- | --- | --- | --- |
| Gender, n(%)  female  male  Age(yr), mean ± SD  Smoking history, n (%)  No  Yes  Alcohol consumption, n (%)  No  Yes  Family history of cancer, n (%)  Disease history, n (%)  Clinical stage, n (%)  I  II  III  IV  T stage, n (%)  T1  T2  T3  T4  N stage, n (%)  N0  N1  N2  N3  M stage, n (%)  M0  M1 | 31(20.67)  119(79.33)  59±10  59(39.33)  91(60.67)  105(70.00)  45(30.00)  9(6.00)  45(30.00)  6(4.00)  32(21.33)  77(51.33)  35(23.33)  11(7.33)  12(8.00)  59(39.33)  68(45.33)  38(25.33)  72(48.00)  36(24.00)  4(2.67)  131(87.33)  19(12.67) | 51(28.81)  126(71.19)  57.47±14.12 | 0.076^a^  0.148^b^ |

^a^ data analysis by Chi-square test；^b^ data analysis by t test
